# Validating the Copenhagen Psychosocial Questionnaire (COPSOQ-II) Using Set-ESEM: Identifying Psychosocial Risk Factors in a Sample of School Principals

**DOI:** 10.3389/fpsyg.2018.00584

**Published:** 2018-04-30

**Authors:** Theresa Dicke, Herbert W. Marsh, Philip Riley, Philip D. Parker, Jiesi Guo, Marcus Horwood

**Affiliations:** Institute for Positive Psychology and Education, Australian Catholic University, Sydney, NSW, Australia; Department of Education, University of Oxford, Oxford, United Kingdom

**Keywords:** COPSOQ-II, psychosocial risk factors, ESEM, school principals, occupational wellbeing, psychometrics, burnout

## Abstract

School principals world-wide report high levels of strain and attrition resulting in a shortage of qualified principals. It is thus crucial to identify psychosocial risk factors that reflect principals' occupational wellbeing. For this purpose, we used the Copenhagen Psychosocial Questionnaire (COPSOQ-II), a widely used self-report measure covering multiple psychosocial factors identified by leading occupational stress theories. We evaluated the COPSOQ-II regarding factor structure and longitudinal, discriminant, and convergent validity using latent structural equation modeling in a large sample of Australian school principals (*N* = 2,049). Results reveal that confirmatory factor analysis produced marginally acceptable model fit. A novel approach we call set exploratory structural equation modeling (set-ESEM), where cross-loadings were only allowed within a priori defined sets of factors, fit well, and was more parsimonious than a full ESEM. Further multitrait-multimethod models based on the set-ESEM confirm the importance of a principal's psychosocial risk factors; Stressors and depression were related to demands and ill-being, while confidence and autonomy were related to wellbeing. We also show that working in the private sector was beneficial for showing a low psychosocial risk, while other demographics have little effects. Finally, we identify five latent risk profiles (high risk to no risk) of school principals based on all psychosocial factors. Overall the research presented here closes the theory application gap of a strong multi-dimensional measure of psychosocial risk-factors.

## Introduction

While teacher strain and consequent attrition have been identified as a worldwide problem (Tsouloupas et al., 2010; Dicke et al., 2015a, 2017), research on school principals' occupational wellbeing is still scarce (Darmody and Smyth, 2016; though see Ilies et al., 2015; Fuller and Hollingworth, 2017). Studies that have focussed on school principals reported high levels of strain leading to high attrition and a shortage of qualified principals (e.g., Dewa et al., 2009; Riley, 2014, 2015, 2017; Grissom et al., 2015; Darmody and Smyth, 2016). This is alarming considering that principal leadership is crucial to a school environment that fosters teachers' wellbeing (Collie et al., 2016) and thus, indirectly (Arens and Morin, 2016; Klusmann et al., 2016) and directly students' learning and wellbeing (Koh et al., 1995; Day, 2011). This implies a need to identify risk factors for school principals and to assist in the development of effective strategies to enhance protective resources and wellbeing, for this at-risk occupational group.

Although research has clearly established a link between psychosocial factors and employee wellbeing, there remains a gap between research and application, particularly with regard to comprehensive measurement instruments (Bailey et al., 2015; Zheng et al., 2015), and their construct validity (Schat et al., 2005). The COPSOQ is a widely used as a tool for psychosocial risk assessment in the workplace and has been used in thousands of enterprise based risk assessments (Nübling et al., 2014). The COPSOQ covers a broad array of important psychosocial factors at work based on the leading concepts and theories of occupational health and wellbeing. It is increasingly being used for research purposes (Nübling and Hasselhorn, 2010). Evaluating the factor structure of instruments such as the COPSOQ, which purposely include multiple related scales (dimensions) of the same domain, requires new statistical models that takes this substantive structure into account. In particular, exploratory structural equation modeling (ESEM) allows relaxation of the unique factor assumption, where each item is hypothesized to load on one and only one factor (Marsh et al., 2009, 2014). However, this technique loses parsimony with an instrument such as the COPSOQ which presupposes logical multiple factor loadings for its dimension level. Thus, a balance of ESEM and parsimony is needed for such instruments. We have developed such an approach, where we allow loadings on multiple factors, but only within theoretically meaningful sets, an approach we name set-ESEM.

The present research is one of the first comprehensive studies of school principals psychosocial risk factors. We first focus on the factor structure and validity of measuring school principals' psychosocial risk factors based on the COPSOQ using an innovative new approach to ESEM (set-ESEM). We can confirm the assumed structure of the COPSOQ which includes 34 distinct factors. Thus, set-ESEM shows better model fit than CFA and comparable model fit to traditional ESEM. Almost all 120 items load more strongly on the set-ESEM factor that they were designed to load on and then on all other factors. Finally, a thorough multi-trait multi-method approach reveals strong evidence for re-test reliability.

Based on the results of the set-ESEM model we examine individual differences in the psychosocial risk factors related to demographic characteristics of school principals. Finally, we identify sub-groups within our sample that show similar risk profiles. Taken together, the present study takes an important step in bridging research and application within the important and understudied field of school principal wellbeing by bringing together new findings of substantive, measurement related, and methodological matters.

### School principals declining health

Changes to the principal role over the previous two decades is the likely cause of a shortage of qualified school leaders due to the attrition of the experienced teaching workforce and an increasing reluctance to “step up” to the role of leader (For an overview see Gates et al., 2006; Miller, 2013). Declining applications is a worldwide concern (Gallant and Riley, 2014). Principals face increasing accountability promoted by the global educational reform movement (GERM: Sahlberg, 2015). A significant stressor in GERM countries has been the increased emphasis by governments on accountability for uniform curriculum delivery along with the devolution of administrative tasks from central to local control. This has led to increased job demands and diminished resources, particularly decreased decision latitude.

More disturbing is that under these conditions younger people appear to be at greater risk of coronary heart disease than their older colleagues (Kuper and Marmot, 2003).

Phillips and Sen (2011) reported that, “work related stress was higher in education than across all other industries… with work-related mental ill-health… almost double the rate for all industry” (pp. 177–178). In addition, retiring principals will be replaced with younger, less experienced individuals who are potentially more at risk of experiencing the negative impacts of the role (Kuper and Marmot, 2003; Riley, 2014, 2015; Darmody and Smyth, 2016). This is particularly alarming considering that leadership is crucial to an effective school environment that fosters students' learning (Day, 2011; Leithwood and Louis, 2012). Put simply, declining school principals' wellbeing leads to a reduced ability to significantly impact school functioning and teacher and student engagement and thus, whole-school wellbeing also declines (Leithwood et al., 2008; Ten Bruggencate et al., 2012; Arens and Morin, 2016; Collie et al., 2016; Klusmann et al., 2016; Maxwell and Riley, 2017). Hence, it is essential to further identify causes and predictors of principals' diminishing occupational health.

### Psychosocial risk factors at work

Cox and Griffiths (2005) define psychosocial risks at work as aspects regarding work design as well as the social, organizational, and management contexts of work that could potentially cause physical or psychological harm. Indeed, the link between occupational psychosocial aspects and mental health has long been established (see Bailey et al., 2015 for an overview). In line with these assumptions many models and theories that are important in stress research (and beyond) focus on the relationship of psychosocial aspects and employee mental health. Kompier in his review (2003; p. 429), identified seven major influential theories “to find the factors in work that affect stress and psychological wellbeing”:

1. The Job Characteristics Model (JCM; Hackman and Oldham, 1976),
2. The Michigan Organizational Stress model (MOS; Caplan et al., 1975),
3. The Demand–Control–(Support) Model (DCM; Karasek, 1979, 1990),
4. The Sociotechnical Approach (Kuipers and Van Amelsvoort, 1993),
5. The Action–Theoretical Approach (Frese and Zapf, 1994),
6. The Effort–Reward–Imbalance model (ERI; Siegrist, 1996), and
7. The Vitamin Model (Warr, 1996; De Jonge and Schaufeli, 1998; Kristensen et al., 2005).

Kompier found that, despite some differences, such as being individually centered (1 and 2) vs. being centered on the environment (3, 4, 5, and 7) or both (6), these theories showed parallels in substantial areas, such as finding very similar determinants of job related well-being, i.e., all models agree on the importance of skill variety, demands, or social support as psychosocial drivers at work (see Kompier, 2003 for details).

### Psychosocial risk factors for school principals

The principal's occupation is multifaceted. It incorporates a wide range of different work elements, including various aspects of leadership, working with policy makers, providing a service to clients (parents and students), financial budgeting, recruitment, strategic projects, reporting, teacher evaluations (Torff and Sessions, 2005) and of course teaching itself (see also Dadaczynski and Paulus, 2015). Moreover, school principals are challenged by a very diverse leadership role where they are required to be visionaries and directors, people developers, organization designers, and teaching and learning program managers (Leithwood, 1994; Dadaczynski and Paulus, 2015). Recent changes in society, such as an increasing globalization, new technologies, and changes in workforce demographics (Stiglbauer, 2017), have led to the education system responding by changing the role of school leaders (Dewa et al., 2009). Thus, principals now have greater responsibility (particularly in managerial issues; Green et al., 2001), higher time pressure (Grissom et al., 2015), less decision latitude, and reduced autonomy (Riley, 2017). In addition, Maxwell and Riley (2017) showed that emotional demands due to the multitude of interactions with parents, teachers, and other stakeholders predicted burnout. These results provide empirical support for Friedman (2002) whose comprehensive review of the literature resulted in a comprehensive list of principal stressors. He showed that ongoing demands such as interpersonal stress sources, including interactions with staff and parents, affected burnout levels in addition to general role overload and administrative constraints (Friedman, 2002; Poirel et al., 2012). These increased demands, but a lack of adequate resources or reward to compensate these consequently lead to the high levels of strain (Riley, 2014, 2015, 2017).

In the present study we thus investigate as stressor covariates: sheer quantity of work, expectations of the employer, student related issues, parent related issues, government initiatives, lack of autonomy/authority, financial management issues, and interpersonal conflicts. Furthermore, we investigate the relationship of relevant COPSOQ dimensions with depression, job related autonomy, and confidence.

### The role of demographic characteristics

As shown above, research on principals' psychosocial risk factors and job-related health and wellbeing are still understudied. While numerous studies contribute to so called “laundry lists” of stressors and demographic differences for teachers (see Dicke et al., 2015b), it is hard to find such studies explicitly for principals (but see, e.g., Friedman, 2002; Dewa et al., 2009; Darmody and Smyth, 2016) and those that do mostly focus on burnout and principal turnover (but see Federici and Skaalvik, 2012). The studies that have investigated such demographic differences in psychosocial risk factors and occupational wellbeing have found, in part, inconsistent results (see also Dadaczynski and Paulus, 2015). While some report female school principals to show higher levels of mental health related problems (Weber et al., 2005; Dadaczynski and Paulus, 2011), others found no such (or only negligible) differences (Friedman, 2002; Dewa et al., 2009; Darmody and Smyth, 2016). Interestingly, most studies find age to be negatively related to mental health, although years of experience seems to be a protective factor (Darmody and Smyth, 2016). School location (e.g., urban vs. rural) had no effect on mental health (Darmody and Smyth, 2016). Studies looking at other psychosocial factors in school principals found no effects of age or gender on self-efficacy or job satisfaction (Federici and Skaalvik, 2012; Darmody and Smyth, 2016), except for Darmody and Smyth (2016) finding a small negative effect of age on job satisfaction. There were also no effects of school location (e.g., urban vs. rural) on job satisfaction (Darmody and Smyth, 2016). However, job satisfaction was negatively correlated to the length of experience a school principal had in the job, showing a decline in job satisfaction over time (Darmody and Smyth, 2016). Darmody and Smyth (2016) also found better quality school facilities to foster job satisfaction. Although they did not explicitly account for school type, better facilities are usually associated with high income or resource schools, such as private (fee paying) schools and are thus heavily related to the socio-economic status of the school population.

Taken together, there is still a need to identify demographics that are predictive of psychosocial factors and wellbeing to identify high risk groups and develop individualized measures for prevention. In the present study, we will examine differences based on gender, age, school location (urban/rural/remote), and school type (public/private).

### Identifying risk profiles

A different approach to examining individual differences is achieved by taking into account different subpopulations in which the observed relations between variables may differ, quantitatively and qualitatively. In these person-centered analyses, as opposed to typical variable-centered approaches that are based on the overall sample mean, it is then possible to identify profiles of these subgroups (Morin et al., 2011; Morin and Marsh, 2015). There is still a paucity of such profiles with regard to occupational wellbeing for educational personnel. Some authors have investigated profiles for teachers (e.g., Klusmann et al., 2008; Collie et al., 2015), but to our knowledge not yet for school leaders. For example, Klusmann et al. (2008), identified four profiles: healthy–ambitious, unambitious, excessively ambitious, and resigned. However, Klusmann et al. (2008) investigated teachers and focused on only two psychosocial factors, namely work engagement and resilience. In order to more fully depict psychosocial risk profiles, it is necessary to include a more comprehensive set of risk factors. This need led us to choose the COPSOQ (Pejtersen et al., 2010b) instrument to investigate these differences.

### The COPSOQ a developing tool for assessing psychosocial risk factors

The COPSOQ was developed as a tool for practice and research (Kristensen, 2010; Nübling et al., 2014) and explicitly states as one of its aims to develop valid and relevant instruments for the assessment of psychosocial factors at work (Kristensen et al., 2005, p. 439).

Theoretically, the original COPSOQ is based on this work by Kompier (2003; see above) and the COPSOQ items have been developed to cover all of the major theories of workplace functioning and thus, the important aspects defined by them.

For the present study we will focus on the redefined long version of the COPSOQ-II instrument (Pejtersen et al., 2010b). The revised survey suggests seven overarching domains based on Kompier's (2003) aforementioned meta-theoretical review, namely “Demands At Work,” “Work Organisation and Job Contents,” “Interpersonal Relations and Leadership,” “Work-Individual Interface,” “Personality,” “Values at the workplace,” and “Health and Wellbeing.” These domains then include several dimensions (Personality only includes one), for example “Work-Individual Interface” consists of the dimensions “Job insecurity,” “Job satisfaction,” “Work–family conflict,” and “Family–work conflict” (see Table 1 for a list of all domains and their dimensions). Thus, the COPSOQ includes important psychosocial workplace dimensions including predictors and outcomes, such as General Health, Burnout, Influence, Trust in Management, and Emotional demands.

**Table 1:** COPSOQ structure, number of items, omegas, and examples of all scales.

**Domain**	**Dimension**	**Abbreviation**	**Omega**	**No. of items**	**Version**	**(Example) Items**
Health and well-being	General health rating	GH	–	1	L,M,S	In general, would you say your health is: Excellent/very good/good/fair/poor
	Burnout	BO	0.91	4	L,M,S	How often have you been emotionally exhausted?
	Stress	ST	0.89	4	L,M,S	How often have you been stressed?
	Troubles sleeping	SL	0.89	4	L,M	How often have you slept badly and restlessly?
	Depressive symptoms	DS	0.81	4	L	How often have you felt sad?
	Somatic stress symptoms	SO	0.71	4	L	How often have you had stomach ache?
	Cognitive stress symptoms	CS	0.87	4	L	How often have you had problems concentrating?
Personality	Self-efficacy	SE	0.80	6	L	It is easy for me to stick to my plans and reach my objectives.
Work-individual Interface	Job insecurity	JI	0.72	4	L	Are you worried about becoming unemployed?
	Job satisfaction	JS	0.82	4	L,M,S	Satisfied with your job as a whole, everything taken into consideration?
	Work-family conflict	WF	0.87	4	L,M,S	Do you feel that your work takes so much of your time that it has a negative effect on your private life?
	Family-work conflict	FW	–	2	L	Do you feel that your private life takes so much of your time that it has a negative effect on your work?
Interpersonal relations and leadership	Job predictability	PR	–	2	L,M,S	Do you receive all the information you need in order to do your work well?
	Job rewards	RE	0.87	3	L,M,S	Does the management at your workplace respect you?
	Role clarity	CL	0.86	3	L,M,S	Do you know exactly what is expected of you at work?
	Role conflicts	CO	0.84	4	L,M	Are contradictory demands placed on you at work?
	Quality of leadership	QL	0.91	4	L,M,S	Your supervisor gives high priority to job satisfaction?
	Social support from colleagues	SC	0.78	3	L,M	How often do you get help and support from your colleagues?
	Social support from supervisor	SS	0.87	3	L,M,S	How often do you get help and support from your nearest superior?
	Social community	SW	0.81	3	L,M	Do you feel part of a community at your place of work?
Demands at work	Quantitative demands	QD	0.83	4	L,M,S	Do you get behind with your work?
	Work pace	WP	0.87	3	L,M,S	Do you have to work very fast?
	Cognitive demands	CD	0.77	4	L	Do you have to keep your eyes on lots of things while you work?
	Emotional demands	ED	0.79	4	L,M,S	Is your work emotionally demanding?
	Demands for hiding emotions	HE	0.66	3	L	Does your work require that you hide your feelings?
Work organization and job contents	Influence	IN	0.74	4	L,M,S	Do you have any influence on what you do at work?
	Possibilities for development	PD	0.80	4	L,M,S	Does your work give you the opportunity to develop your skills?
	Variation	VA	–	2	L	Is your work varied?
	Meaning of work	MW	0.85	3	L,M,S	Is your work meaningful?
	Commitment to the workplace	CW	0.77	4	L,M,S	Do you enjoy telling others about your place of work?
Values at workplace level	Trust in management	TM	0.75	4	L,M,S	Can you trust the information that comes from the management?
	Mutual trust between employees	TE	0.77	3	L,M	Do the employees in general trust each other?
	Justice	JU	0.85	4	L,M,S	Is the work distributed fairly?
	Social responsibility	SI	0.80	4	L	Is there space for employees of a different race and religion?

*L, Long version; M, Medium version; S, Short version*.

The COPSOQ questionnaire has been translated into over 25 languages to date (Berthelsen et al., 2016). Several studies have validated these (translated) COPSOQ versions, finding minimally important differences within scales (Pejtersen et al., 2010a) and reliability (Thorsen and Bjorner, 2010). Furthermore, construct validity has been tested by examining differential item functioning (Bjorner and Pejtersen, 2010) and exploratory analyses of the factor structure, ceiling, and floor effects (Moncada et al., 2014). Further, some studies have focused on the predictive quality of COPSOQ scales for important occupational outcomes, such as vitality and mental health (Burr et al., 2010), sickness absence (Clausen et al., 2012; Olesen et al., 2012; Rugulies et al., 2016), and affective organizational commitment (Clausen and Borg, 2010). These studies have mostly applied analyses based on manifest scale scores, not taking measurement error into account and rely on CFA like factor structures of the COPSOQ-II dimensions, i.e., where every item loads on one dimension and one dimension only. For a list of review articles see the COPSOQ International Network website (https://www.copsoq-network.org/validation-studies/).

To date, however, there is no published study that has juxtaposed the specific factor structures (construct validity), including discriminant, and convergent validity of the COPSOQ using more appropriate SEM methods. Further, the COPSOQ instrument is frequently used for assessing changes in psychosocial variables (e.g., Clausen and Borg, 2010; Nübling et al., 2013) and purposes such as improvement for working conditions (Kristensen, 2010), all of which require pre-and post-measures of the same instrument. Nevertheless, its convergent and discriminant validity in relation to stability over time (re-test reliability) has not sufficiently been tested, particularly in a comprehensive matter including all dimensions and their interrelations.

### The present study

Utilizing a large sample of an at risk occupational group, namely school principals, the aim of the present study is to identify and closely examine individual differences in important psychosocial risk factors for this at -risk occupation. Further, we validate the COPSOQ-II instrument which, although widely used in applied settings (Kristensen, 2010; Nübling et al., 2014), has not been psychometrically scrutinized using state of the art methodology.

Thus, as a prerequisite to any substantive research questions we will first validate the COPSOQ-II in our sample of school principals; i.e., we will (a) investigate the factor structure of the COPSOQ-II and (b) test for re-test reliability of these scales.

Next we will validate the importance of the psychosocial risk factors by examining convergent and divergent validity with external scales based on crucial principal covariates. For choosing such substantively important external criteria we have defined variables (see Table 2) that play a role in the development of principals' occupational wellbeing based on our literature review of empirical findings (e.g., Carr, 1994; Caruso et al., 2004; Riley, 2017) and theoretical models of stress research e.g., the ERI (Siegrist, 1996), DCM (Karasek, 1979), MOS (Caplan et al., 1975). Thus, we define a priori with which COPSOQ-II scale these covariates will correlate highest (see Table 2 which includes references for the proposed relationships) and utilize a multitrait-multimethod model (MTMM) for testing our assumptions.

**Table 2:** A-priori prediction of covariates' highest correlations with the COPSOQ.

**Covariate**			**Highest correlate**	
**Topic**	**Item**	**References**	**Domain**	**Dimension(s)**
Stress sources	*Sheer quantity of work*	Green et al., 2001	Demands at Work	Quantitative Demands
	*Expectations of the employer*	Leithwood, 1994	Interpersonal Relations and Leadership	Rewards
	*Student related issues*	Friedman, 2002	Demands at Work	Emotional demands
	*Parent related issues*	Friedman, 2002	Demands at Work	Emotional demands
	*Financial management issues*	Poirel et al., 2012	Interpersonal Relations and Leadership	Role conflicts
	*Inability to get away from school/community*	Riley, 2014	Work-individual Interface	Work-family conflict
	*Interpersonal conflicts*	Friedman, 2002	Interpersonal Relations and Leadership	Trust in Management
Depression	*I am frequently depressed about my job*.	Carr, 1994	Health And Well-Being	Depressive Symptoms
Autonomy	…*in providing strategic focus and direction to colleagues*	Riley, 2017	Work Organization And Job Content	Influence
Confidence	…*in providing strategic focus and direction to colleagues*	Darmody and Smyth, 2016	-	Self-Efficacy

Then, we examine individual differences due to demographic characteristics in these factors. Finally, we will identify subgroups within our sample and will determine risk profiles of these sub groups.

#### Hypotheses and research questions:

- ***Hypothesis 1***:
  ◦ *a:* The factor structure of the COPSOQ-II dimensions will satisfactorily fit when constrained to a CFA model, but will provide better fits, with regard to a priori defined cut-off criteria, when using less restrictive modeling approaches (ESEM), while maintaining the intended loading structure.
  ◦ *b:* The COPSOQ-II dimensions will show strong convergent and discriminant validity in relation to stability over time (i.e., using time as the method variable in a multitrait-multimethod analysis; Campbell and Fiske, 1959; Marsh, 1988).
- ***Hypothesis 2***: Typical school principals' occupational wellbeing variables will have high convergent and discriminant validity in relation to matching COPSOQ-II scales (see Table 2), i.e., the convergent relationships to be high and higher than the relationships of the wellbeing variables with non-matching (divergent) COPSOQ-II scales. Overall, we expect stress sources to correlate with demands and/or indicators of ill-being, while we expect autonomy and confidence to correlate highest with personal resources and well-being (e.g., Friedman, 2002; Poirel et al., 2012; Maxwell and Riley, 2017).
- ***Hypothesis 3***: Although results regarding gender have been inconsistent, we still expect female or older principals to experience higher levels of those dimensions reflecting ill-health and demanding factors (Dewa et al., 2009), but no such effects for school type or school location (Darmody and Smyth, 2016). We expect school principals that work at private schools or school principals that have recently started (Darmody and Smyth, 2016) to report higher job satisfaction. We leave as a research question any other effects of demographic differences on psychosocial risk factors.
- ***Research Question 1***: This study is one of the first studies to investigate comprehensive school principals risk profiles (person-centered approach) and due to the lack of existing literature, is essentially exploratory. Thus, we leave as an open question as to how many risk profiles and the nature of these profiles.

## Methods

### Participants

Participants were school principals working in Australia during 2011 (and partly in 2012). The sample (*N* = 2,049) comprised 44.4% male and 55.6% female participants: 70.3% principals, 25.4% % assistant/deputy principals, and 3.3% campus principals of a multi-campus school. The mean age of school principals in our sample was 57.61 (*SD* = 7.29) years. Regarding the school types the principals managed, 64.0% were primary schools, 22.0% were secondary schools, and 13.9% were combination schools (both primary and secondary). The mean years of experience in their current position was 5.2 years (Min. 0 and Max. 41 years) and 12.5 years in leadership roles generally (Min. 0 and Max. 42 years).

Data on these school principals, were collected as part of a large research project on principal health and wellbeing (Riley, 2014, 2015, 2017), where principals filled in a large survey annually. In the present study, we focus on data assessed at the first time wave of this larger project collected in 2011, but additionally use data from the second wave (in 2012) to test our second hypotheses. Missing data was assumed to be missing at random and handled using the full information maximum likelihood (FIML) approach (Enders, 2010).

### Measures

We focused on the COPSOQ-II dimensions that consisted of the Likert scaled item types (ranging on a “strongly agree” to “strongly disagree” continuum (which excluded the Offensive Behavior items)). Thus, we included 34 scales from the long version. For an overview of all scales (included in each version examined in the present study), example items, number of items, and internal consistency, see Table 1. McDonald's Omega (1999), which reflects the proportion of variance in the scale scores accounted for by a general latent factor, is reported as a measure of internal consistency (see also Zinbarg et al., 2006; for alpha values and confidence intervals for both, omegas and alphas see Supplementary Material). In general, Omega coefficients were 0.79 on average. All scales showed omega values above 0.7, except for Hiding Emotions (0.66).

*Covariates*. We included several other typical principals' wellbeing variables as covariates. These included several stress sources that are typical for school principals (i.e., Sheer Quantity of Work, Expectations of the Employer, Student Related Issues, Government Initiatives, Parent Related Issues, Lack of Autonomy/Authority, Financial Management Issues, and Interpersonal Conflicts) as well as three items to assess school principals' level of depression, job related autonomy, and confidence (see Table 2).

### Analysis

In a first step we examined the factor structure of the COPSOQ-II. Researchers typically use CFA models for validation of such factor structures. In the present study however we also use ESEM. The difference between the CFA and ESEM approaches is that in ESEM all factor loadings are estimated, excluding those constraints necessary for identification (see Asparouhov and Muthén, 2009; Marsh et al., 2009, 2013). Although there are many methodological and strategic advantages to CFAs, these models typically do not provide an acceptable fit to the data (Marsh et al., 2010a). This is most likely due to the overly restrictive assumption of CFA (each item is hypothesized to load on to only one factor), and the misspecification of factor loadings (automatically constraining them to be zero), which usually leads to distorted factors with over-estimated factor correlations (Marsh et al., 2013). These positively-biased factor correlations can lead to biased estimates in SEMs incorporating other outcome variables (Asparouhov and Muthén, 2009; Marsh et al., 2009; Schmitt and Sass, 2011).

ESEM, however, not only provides a better fit, but also results in latent factors that are more differentiated (i.e., less correlated; Asparouhov and Muthén, 2009) and accurately estimated. This results from ESEM using two estimates of overlap between factors (overlap in factor loadings and correlation between factors), compared to CFA, which uses one estimate (correlation between factors; Marsh et al., 2010a). In our study we will use ESEM with target rotation (for details see Browne, 2001; Asparouhov and Muthén, 2009). Despite its advantages when compared to CFA, ESEMs with a large number observed indicators are naturally not very parsimonious and can lead to convergence problems (due to computational issues). Therefore, we also tested an alternative set-ESEM model where all indictors are only allowed to cross-load within an a priori defined set of factors based on a theoretically meaningful structure. In case of the present study, the specified cross-loadings were based on the suggested implicit overarching domains of the COPSOQ-II (Pejtersen et al., 2010b). These cross loadings within sets are then constrained to be close to zero, while cross-loading between sets are constrained to be zero.

Generally, given the known sensitivity of the chi-square test to sample size, minor deviations from multivariate normality, and minor misspecifications, applied SEM research focuses on indices that are relatively sample-size independent (Hu and Bentler, 1999; Marsh et al., 2004) such as the Root Mean Square Error of Approximation (RMSEA), the Tucker-Lewis Index (TLI), and the Comparative Fit Index (CFI). Population values of TLI and CFI vary along a 0-to-1 continuum, in which values > 0.90 and 0.95 typically reflect acceptable and excellent fits to the data, respectively. Values smaller than 0.08 and 0.06 for the RMSEA show acceptable and good model fits.

As is recommended for self-report surveys that include a mixture of positively and negatively worded items, we specified correlated uniquenesses (CUs) relating the responses to each of the negatively worded items (e.g., Marsh, 1996; Marsh et al., 2010c) for avoiding method effects associated item wording (Marsh et al., 2010a) and consequent bias.

The multitrait-multimethod model (MTMM) design, we apply in the current study, is widely used to assess convergent and discriminant validity and is one of the standard criteria for evaluating psychological instruments (e.g., Campbell and Fiske, 1959; Marsh, 1988; Marsh et al., 1994). Campbell and O'Connell (1967) and Marsh et al. (2010b) showed how operationalizing the multiple methods in their MTMM paradigm across multiple occasions provides a very strong approach to evaluating stability of responses to a multidimensional instrument.

Thus, we compare convergent validities (correlations between matching traits—test-retest correlations when method is based on time), heterotrait-heteromethod (correlations between different traits measured on different occasions), and heterotrait-homomethod correlations (correlations among nonmatching traits collected on the same occasion). Convergent validity is supported when convergent validates (correlations) are high. Discriminant validity, on the other hand, is supported when convergent validities are larger than all other correlations. We infer method effects in case heterotrait-homomethod correlations involving a particular method are higher than heterotrait-heteromethod correlations or approach 1.0.

We used the model-based clustering MCLUST package (Fraley et al., 2014; Scrucca et al., 2016) in R (R Core Team, 2014) for identifying latent risk profiles by means finite Gaussian mixture modeling fitted via EM algorithm. Thus, we assumed that correlations between the indicators, i.e., for the present research the psychosocial risk factors, can be explained by an underlying latent categorical cluster variable representing qualitatively and quantitatively distinct profiles of principals within the sample population (Morin et al., 2011). We identified the best fitting model solution with regard to number of profiles and covariance structure based on the Bayesian information criterion (BIC) and integrated complete-data likelihood criterion (ICL; Scrucca et al., 2016). For evaluating our profile solution we used probabilistic discriminant analysis (MclustDA; Fraley and Raftery, 2007), where a model is fit to each class in the training set. Observations of a test set are then assigned to the class corresponding to the model in which they have the highest posterior probability (Fraley and Raftery, 2007). It is then possible to estimate errors for the training and the test data as well as a cross-validation error.

## Results

### Descriptives

The latent correlations of all factors based on the CFA, ESEM, and set-ESEM are reported in Table SI1-SI3 (see Supplementary Material and online for better readability of Tables SI1-SI5 and SA1-SA16 https://figshare.com/s/c69bde7bbd73e8de70f2).

### Factor structure of the COPOSOQ-II (hypothesis 1a)

For examining the first-order factor structure of the COPSOQ-II long version we compared three different models: (1) a CFA, where cross-loadings are constrained to be zero, (2) ESEM with target rotation, were cross-loadings are allowed for all other factors, and (3) a set-ESEM approach with target rotation, were cross-loadings are only allowed within a defined set of factors. (For results of these tests regarding the medium and short version of the COPSOQ-II please see additional Supplementary Material Annex Tables SA1–7).

#### CFA model

The CFA model fit the data satisfactory (χ^2^ = 18,854, *df* = 6,445, CFI = 0.907, TLI = 0.898, RMSEA = 0.031) with CFI and TLI being minimally acceptable. However, correlations (Mean [*M*] | *r* | = 0.289) between some factors were very high with values up to | *r* | = 0.881 and 10% of the correlations had absolute values above 0.50 (see Supplementary Material Table SI1). These high correlations may be evidence that a CFA model is not appropriate and overly restricted which causes inflated factor correlations.

#### ESEM

When running the model as an ESEM with target rotation, model fit improved substantially (χ^2^ = 10,976, *df* = 3,621, CFI = 0.971, TLI = 0.94, RMSEA = 024). Correlations dropped (*M* | *r* | = 0.205) with the highest | *r* | = 0.77 and only 5% of the correlations had absolute values above 0.50 (see Supplementary Material Table SI2). This model, however, has a large number of estimated parameters (*df* = 3621) as cross loadings are allowed for all factors in the model and thus lacks parsimony.

#### Set-ESEM

The set-ESEM approach with target rotation provides a method to take advantage of EFA-like structures, thus providing a more realistic model by taking into account the a priori factor structure, but being more parsimonious (χ^2^ = 12,669, *df* = 5,987, CFI = 0.947, TLI = 0.938, RMSEA = 023) as cross-loadings are only allowed within a priori defined set of factors. Model fit remained good and was not substantially different than the ESEM model with regard to cut-off values (ΔCFI < 0.01, ΔTLI < 0.01, ΔRMSEA < 0.015). In fact, those fit indices that control for parsimony remain almost unchanged (0.94 to 0.938 for TLI;0.024 to 0.023 for RMSEA). And indeed, the χ^2^ difference test showed a non-significant difference between the full ESEM and ESEM-set for the long version of the COPSOQ-II, further indicating the appropriateness of the set-ESEM in line with our expectations (H1).

Three scales were excluded from this set-ESEM structure and included as CFAs: (1) The Self-Efficacy construct is the only dimension in the personality domain, (2) The General Health one-item scale was excluded for reasons of model identification, and (3) the two item Variation scale was modeled as a CFA as it showed very high unsystematic cross-loadings. On average, correlations of this set-ESEM model (*M* | *r* | = 0.263) were slightly higher than those of the ESEM model, however, the highest correlation was lower with |*r* | = 0.760 and in this model also only 5% of the correlations were above absolute 0.50 (see Supplementary Material Table SI3).

#### Factor loadings

The factor loadings for the CFA model are slightly higher than those of the ESEM and set-ESEM models (*M* = 0.736, *M* = 0.638, and *M* = 0.678 respectively) but reveal only small differences (see Supplementary Material annex Tables SA8–10). The higher loadings within the CFA model were expected due to the highly constrained nature of CFA models (Asparouhov and Muthén, 2009; Marsh et al., 2009). Importantly, almost all 120 items load more strongly on the set-ESEM factor that they were designed to load on Pejtersen et al. (2010b) and then on all other factors.

Overall, the pattern of results for the long COPSOQ-II version were largely replicated for the medium and short version of the instrument (see Supplementary Material annex Tables SA1–7).

### Re-test reliability (hypothesis 1b)

It was not possible to model the set-ESEM for two time waves simultaneously for testing longitudinal invariance due to inadequate memory and computing capacity of statistical programs to process the large number of factors and manifest indicators. Thus, for investigating the convergent and discriminant validity in relation to stability over time of the instrument we derived factor scores (based on the best fitting ESEM-set model) of the long version of the COPSOQ-II at T1 and T2 (one year later). We then utilized a MTMM-like approach. In the classic MTMM model, two or more traits are collected by two or more methods. In the present study the traits will be represented by COPSOQ-II dimensions, while method variation is reflected by different time waves. In the matrix, we use 66^1^ scales (33 dimensions on 2 occasions). We then evaluated the pattern of relations among the 33 latent constructs to assess convergent and discriminant validity. The 33 convergent validities (those coefficients shaded in gray in Supplementary Material Table SI4) represent correlations between the same dimensions assessed by different methods (monotrait–heteromethod correlations or test–retest stabilities when the multiple methods are the different occasions). These convergent validities were consistently substantive with a mean of | *r* | = 0.607 (*SD* = 0.291). The correlations between different dimensions assessed on different occasions (heterotrait–heteromethod correlations in the framed square submatrix (Table SI4), not including the convergent validities are substantially lower than the convergent validities with a mean of | *r* | = 0.235 (*SD* = 0.124). In addition, the correlations between different dimensions administered on the same occasion (heterotrait–monomethod correlations in the triangular submatrices) are only slightly larger than the heterotrait– heteromethod correlations, with a mean value of | *r* | = 0.302 (*SD* = 0.168).

Thus, with regard to the original Campbell and Fiske (1959) criteria, we provided strong support for convergent validity (the substantive convergent validities) and strong support for discriminant validity (heterotrait–monomethod and heterotrait–heteromethod correlations that are substantively smaller than the convergent validities). We, however, found some small amount of method effect (heterotrait–monomethod correlations higher than heterotrait–heteromethod correlations) which was associated with the specific occasion of data assessment.

### Correlations with important principal variables: convergent and discriminant validity (hypothesis 2)

For evaluating convergent and discriminant validities with external criterion, we again made use of the MTMM approach. Thus, we correlated all COPSOQ-II dimensions with all covariates (i.e., typical stress sources, autonomy, depression, and confidence) based on our latent set-ESEM model. For each criterion, the COPSOQ-II dimension to which it should be most highly correlated was predicted a priori (see Table 2 for an overview of a-priori hypothesis of relationships of these covariates with the COPSOQ dimensions and in Table 3 shaded in gray). Similar to Hypothesis 1, an evaluation of the construct validity of the COPSOQ-II responses follows the logic of MTMM analyses. In this model, (a) support for the convergent validity of the COPSOQ-II responses requires that each predicted correlation is statistically significant, and (b) support for the discriminant validity of the COPSOQ-II responses requires that each external validity criterion is more highly correlated with the predicted COPSOQ-II dimension than any of the other COPSOQ-II dimension.

**Table 3:** Correlations of covariates with the COPSOQ scales.

	**Sheer quantity of work**	**Expectations of the employer**	**Student related issues**	**Parent related issues**	**Financial management issues**	**Inability to get away from school**	**Inter-personal conflicts**	**Depress**.	**Autono**.	**Confid**.
General health rating	−0.215	−0.208	−0.112	−0.122	−0.135	−0.202	−0.156	−0.297	0.12	0.114
Burnout	0.281	0.25	0.147	0.155	0.169	0.246	0.188	0.361	−0.054	−0.051
Stress	0.433	0.309	0.201	0.241	0.217	0.337	0.249	0.432	−0.097	−0.095
Troubles sleeping	0.404	0.37	0.237	0.283	0.267	0.374	0.332	0.543	−0.122	−0.153
Depressive symptoms	0.212	0.327	0.221	0.248	0.198	0.278	0.335	0.6	−0.195	−0.289
Somatic stress symptoms	0.269	0.254	0.156	0.216	0.183	0.283	0.227	0.393	−0.095	−0.074
Cognitive stress symptoms	0.293	0.254	0.173	0.189	0.17	0.256	0.269	0.426	−0.181	−0.208
Self-efficacy	−0.11	−0.138	−0.138	−0.182	−0.115	−0.137	0.182	0.294	0.204	0.449
Job insecurity	0.121	0.237	0.117	0.185	0.137	0.242	0.177	0.35	−0.158	−0.159
Job satisfaction	−0.292	−0.388	−0.214	−0.224	−0.233	−0.281	−0.282	−0.596	−0.318	−0.214
Work-family conflict	0.541	0.341	0.198	0.248	0.259	0.378	0.235	0.374	−0.079	−0.093
Family-work conflict	0.064	*0.036*	0.056	0.055	0.092	*0.018*	0.051	0.099	−*0.01*	−*0.044*
Job predictability	−0.254	−0.325	−0.17	−0.177	−0.215	−0.212	−0.175	−0.307	0.256	0.075
Job rewards	−0.238	−0.411	−0.154	−0.184	−0.203	−0.207	−0.2	−0.38	0.198	0.098
Role clarity	−0.133	−0.181	−0.121	−0.105	−0.111	−0.18	−0.197	−0.32	0.358	0.248
Role conflicts	0.311	0.377	0.234	0.295	0.324	0.288	0.385	0.378	−0.105	−0.061
Quality of leadership	−0.173	−0.259	−0.056	−0.071	−0.142	−0.137	−0.114	−0.237	0.146	*0.008*
Social support from colleagues	−0.141	−0.189	−0.13	−0.143	−0.115	−0.222	−0.167	−0.285	0.205	0.132
Social support from supervisor	−0.187	−0.277	−0.083	−0.067	−0.107	−0.084	−0.116	−0.195	0.09	−*0.02*
Social community	−0.063	−0.122	−0.141	−0.168	−0.1	−0.193	−0.363	−0.316	0.214	0.248
Quantitative demands	0.553	0.318	0.203	0.195	0.214	0.261	0.206	0.304	−0.089	−0.136
Work pace	0.399	0.221	0.113	0.14	0.109	0.18	0.095	0.168	−*0.03*	0.078
Cognitive demands	0.265	0.216	0.117	0.152	0.187	0.191	0.126	0.121	0.158	0.212
Emotional demands	0.383	0.339	0.335	0.363	0.312	0.279	0.333	0.414	−*0.022*	−*0.006*
Demands for hiding emotions	0.261	0.249	0.173	0.232	0.152	0.22	0.247	0.256	−*0.022*	*0.02*
Influence	−0.315	−0.343	−0.195	−0.181	−0.12	−0.202	−0.197	−0.335	0.396	0.225
Possibilities for development	−0.087	−0.162	−0.125	−0.116	−0.032	−0.15	−0.118	−0.298	0.29	0.253
Meaning of work	*0.054*	−*0.052*	−0.077	−0.069	*0.03*	−*0.049*	−0.1	−0.277	0.321	0.262
Commitment to the workplace	−0.291	−0.319	−0.231	−0.288	−0.192	−0.32	−0.264	−0.57	0.113	0.102
Variation	−*0.022*	−0.119	−0.093	−0.072	-*0.015*	−0.128	−0.09	−0.241	0.202	0.136
Trust in management	−0.102	−0.181	−0.148	−0.164	−0.167	−0.203	−0.376	−0.33	0.206	0.14
Mutual trust between employees	−0.11	−0.239	−0.106	−0.101	−0.094	−0.136	−0.188	−0.249	0.203	0.091
Justice	−0.082	−0.179	−0.121	−0.131	−0.051	−0.123	−0.211	−0.309	0.28	0.169
Social responsibility	−*0.01*	−0.048	−0.057	−0.101	−0.067	−0.088	−*0.086*	−0.121	0.096	0.11

*A-priori prediction of covariates highest correlations with the COPSOQ scales are shaded in gray. Insignificant (p >0.05) correlations are in italic font. (see additional figure file)*.

The analysis model was based on the long set-ESEM model at Time 1 and revealed good fit with χ^2^ = 1,4382, *df* = 7,206, *p* < 0.001, CFI = 0.95, TLI = 0.94, RMSEA = 0.02. Overall results revealed a correlation pattern mostly in line with our expectations (H2). While the matching correlations showed an average of | *r* | = 0.419 (*SD* = 0.087), the non-matching only showed an average of | *r* | = 0.193 (*SD* = 0.104; for details see Table 3). In the summary below we focus on the highest correlations only (for a more detailed overview, see Supplementary Material). Namely, results showed:

#### Stress sources

Based on our literature review we examined correlations with several typical stress sources of school principals.

The *sheer quantity of work* item as expected correlated significantly highest with Quantitative Demands (*r* = 0.553). Further, it correlated moderately to highly with several negative health outcomes, such as Perceived Stress (*r* = 0.433) and Sleeping Troubles (*r* = 0.404) and with Work-Family Conflict (*r* = 0.541).

As hypothesized *Expectations of the employer* correlated highest and negatively with Rewards (*r* = −0.411). It also correlated with Stress, Sleeping Troubles, and Depression (*r* = 0.309, *r* = 0.337, and *r* = 327 respectively). Furthermore, it correlated moderately with Job Satisfaction (*r* = 0.388) and Work-Family Conflict (*r* = 0.341). Other notable correlations where the ions Quantitative Demands (*r* = 0.553) and Emotional Demands (*r* = 0.333). Excessive expectations are related to other high demands as well as a feeling of reduced recognition by the employer, less job satisfaction, higher work-family conflict and consequently higher levels of ill-being.

*Student related issues* correlated moderately to highly with Emotional Demands (*r* = 0.335). *Parent related issues* correlated moderately to highly with Emotional Demands (*r* = 0.363). These interpersonal issues thus, seem to reflect emotionally demanding stressors.

*Financial management issues* correlated as expected highest with Role Conflicts (*r* = 0.324). Other notable correlations showed with Emotional Demands (*r* = 0.313).

*Inability to get away from the school* correlated as expected highest with Work-family conflict (*r* = 0.378). *Interpersonal conflicts* in contrast to our expectations showed its highest correlation (negative) with Role Conflicts (*r* = −0.384), but closely followed by Trust in Management (*r* = 0.376). Additionally, it correlated moderately to highly with Sleeping Troubles (*r* = 0.332), Depressive Symptoms (*r* = 0.335) and Emotional Demands (*r* = 0.333). Conflicts on the interpersonal level are related to role conflicts, less trust and several indicators of ill-being.

Overall, most of these results confirmed convergent and divergent validity in line with our expectations (H3).

#### Depression, autonomy, and confidence

*Depression* in line with our a priori assumptions, correlated highest with Depressive Symptoms (*r* = 0.60), which indicated convergent and divergent validity. However, depression correlated highly with several psychosocial factors (see Table 3 and Supplementary Material), making it an important outcome variable for school principals.

*Autonomy* as hypothesized correlated highest with Influence (*r* = 0.396). Further it showed moderate correlations with Role Clarity (*r* = 0.358), Job Satisfaction (*r* = 0.318), and Meaning Of Work (*r* = 0.321). Autonomy is related to several important occupational wellbeing indicators making it an important resource for principals.

*Confidence*, only correlated significantly with Self-Efficacy (*r* = 0.449), which also provided strong evidence for convergent and divergent validity. Therefore, we could again show convergent and divergent validity of the COPSOQ-II (H3).

### Demographic variables as predictors (hypothesis 3)

We included several demographic variables as predictors of the psychosocial risk factors as identified by the COPSOQ-II. Again, the analysis model was based on the long set-ESEM model at Time 1 and revealed good fit with χ^2^ = 14,531, *df* = 6,948, *p* < 0.001, CFI = 0.941, TLI = 0.929, RMSEA = 0.017. Results revealed a correlation pattern partly in line with our expectations (H3). In the description, here we focus on significant coefficients larger than one (|β ≥ 0.1|; for details and more results see Table SI5).

#### Gender

While women reported higher levels of negative indicators-Somatic stress symptoms (β = −0.179), and Work pace (β = −0.115), Cognitive Demands (β = −0.102), showing that they perceived higher demands, they also reported higher levels of positive indicators, Possibilities for development (β = −0.123), Variation (β = −0.106), and Commitment (β = −0.132).

#### Leadership experience

School leaders with higher levels of experience reported lower levels of Job predictability (β = −0.103), Quality of Leadership (β = −0.129), and particularly Social Support (β = −0.222). In addition, higher levels of experience positively predicted the level of Role conflicts (β = 0.106), and unexpectedly cognitive and emotional demands (β = 0.114 and β = 0.111), respectively.

#### School location

The location (urban/suburban/large town/rural/remote) of the school only predicted Mutual Trust between employees, where school leaders in rural areas reported higher levels (β = 0.105) in comparison to their urban counterparts.

#### School sector

Overall, school principals working at private (vs. public) schools seemed to be better of reporting higher levels of Job Satisfaction (β = 0.115; as expected), Job Predictability (β = 0.138), Job Rewards (β = 0.173), Social Support (β = 0.13), and Influence (β = 0.143), while reporting lower levels of Role Conflicts (β = −0.147) and Demands for Hiding Emotions (β = − 0.105).

#### Age

In Contrast to our expectations older school leaders seemed to thrive as they reported lower levels of Burnout (β = −0.212), Stress (β = −0.177), Cognitive Stress (β = −0.106), Cognitive Demands (β = −0.103), and Demands for hiding Emotions (β = −0.105). Additionally, they reported higher levels of Job Predictability (β = 0.138), Role Clarity (β = 0.128), Meaning of Work (β = 0.165), and Justice (β = 0.139).

Taken together, being older and working at a private school seemed to be of advantage for most psychosocial risk factors, while gender revealed inconclusive results, and experience did not protect, but rather was a disadvantage with regard to psychosocial risk factors.

### School principal's risk profiles (research question 1)

**Figure 1:**
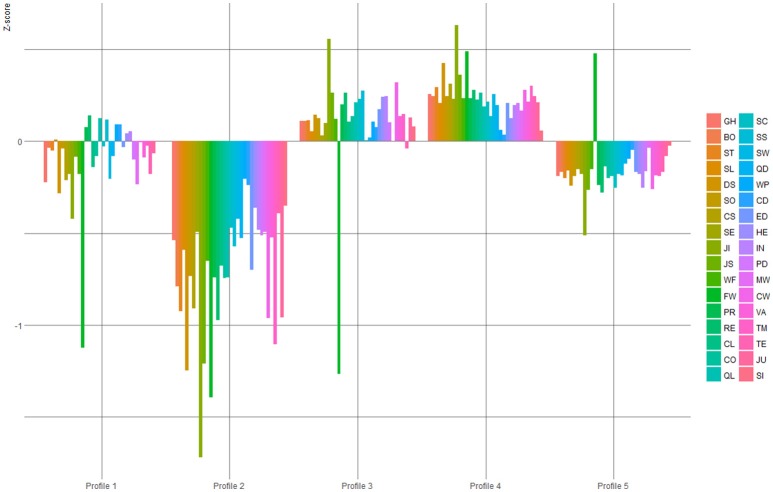
The five-profile solution based on finite Gaussian mixture modeling (Fraley et al., 2014). See Table 1 for a list of scale Abbreviations. For an alternative depiction see Supplementary Material.

Next we applied model based cluster analysis for identifying the latent risk profiles based on the 34 COPSOQ-II factor scores. The results of these risk profiles are shown in Figure 1 (see Supplementary Material for an alternative depiction). The best fitting model (fixed variances and constrained covariances) showed a five-profile solution based on the BIC = −125,874 and the ICL = −126056.6. We confirmed this solution by means of a discriminant analyses, where the profile solution on the whole sample served as known classes. Analyses of test and training data revealed very small errors (0.01 and 0.03, respectively) demonstrating a reliable profile solution.

The distribution of participants (*N*: Profile 1 = 191, Profile 2 = 153, Profile 3 = 227, Profile 4 = 852, and Profile 5 = 626) in profiles showed that the majority (72%), of school principals were either in Profile 4 or 5. The profiles overall revealed level effects rather than shape effects (see Figure 1). Indeed, participants in Risk Profile 2 showed values clearly below the mean (high risk profile), Profile 5 showed values majorly below the mean (moderate risk profile), Risk Profile 1 fluctuating around the mean (average risk profile), Risk Profile 3 majorly above the mean (low risk profile), and Risk Profile 4 clearly above the mean (minimal Risk profile). Two risk factors however stand out in this five-profile solution, namely Job Insecurity and Family Work conflicts. Job Insecurity seems to be one of the most extreme values in all profiles in line with the direction of most risk factors. Family work conflict however, stands out not just because it is also an extreme value in most profiles, but for two profiles (3 and 5) it is additionally in opposite direction of most other risk factors.

## Discussion

We evaluated the psychometric properties of the COPSOQ-II using a sample of Australian school principals. Results revealed that the dimensional structure of the COPSOQ-II holds up well in CFA and ESEM-set. Further, the COPSOQ-II showed high re-test reliability as well as convergent and divergent validity in relation to covariates that play a key role for school principals.

### Factor structure of the COPSOQ (H1a)

Although the good psychometric properties of the COPSOQ-II have so far been successfully validated with regard to different cultures, mixed occupations, and aspects of validity (see Kristensen, 2010 and Berthelsen et al., 2016 for an overview), validation studies based on state of the art methodology such as SEM were still missing. Our approach allows for not only measurement error and differences in item loadings to be taken into account, but also provides more flexibility with regard to model constraints. Thus, even though the CFA model including all dimensions (of that particular COPSOQ-II version) fit the data acceptable, those models where we relaxed overly restricted constraints of items only loading on one factor, fit the data even better, in line with similar studies in research on personality traits that revealed cross-loadings between traits to be a better reflection of reality (see Marsh et al., 2013 for an overview). Applying exploratory approaches to models with a large number of dimensions and consequently items, such as the COPSOQ-II, however, results in very complex models with a large number of estimated parameters which require very high computational and processing capacities. The innovative approach of the present study was in using a more parsimonious set-ESEM approach, where we only allowed cross-loadings between dimensions of the same implicit domain structure. Model fit was very similar compared to the full ESEM and hence, better than the CFA model. This is noteworthy, as the set-ESEM has a strong confirmatory basis if there is good justification for the sets. Indeed, set-ESEM could be placed as midway between CFA and ESEM (with target rotation), depending on the number of factor loadings constrained to be zero. The aforementioned pattern of results (set-ESEM fitting similar to ESEM and better than CFA) emerged for all three COPSOQ-II versions. Despite the only modest differences in fit between the CFA and both ESEM models, at least for the short version of the COPSOQ-II, our findings suggest the ESEM solution to be more appropriate, as it decreased the inflated correlations of closely related dimension to an acceptable level. Additionally, Chi^2^ difference tests showed a non-significant difference between the full and ESEM-set models for the long version of the COPSOQ-II. This indicates that the set-ESEM approach is particularly beneficial in case of very high model complexity. This ESEM-set model should be confirmed in other samples, albeit we consider our results a strong indicator for the models suitability in future research with the COPSOQ-II scales, particularly when considering several theoretically closely related scales simultaneously.

Regarding test-score reliability of the scales we can report omega values above 0.7 for all scales, except for Hiding Emotions (0.66). However, the reliability coefficients are dependent on the number of items per scale (Marsh et al., 1998) and one feature of the COPSOQ is to provide a large number of divers scales, thus, including less numbers of items per scale. Further results of our MTMM analyses provide strong evidence for re-test reliability (see below).

Factor loadings are high for the CFA model with exception of the Variation factor which shows a weak factor loading for one item. This scale should thus, be closely examined in future research. More importantly however, in the more appropriate ESEM-set model (and the full ESEM model) target loadings were always higher than cross-loadings, indicating a good representation of factors through their indicators (Marsh et al., 2013).

Overall, we can report good model fit for all three versions of COPSOQ-II, thus confirming Hypothesis 1a(H1a). Therefore, the instrument offers flexibility for using it in research and the applied context and also offers the user a choice between using different levels of comprehensibility and therefore length.

### Re-test reliability (H1b)

Our results showed strong evidence for convergent and discriminant validity in relation to stability over time of the COPSOQ-II dimensions in line with other studies that have investigated the COPSOQ-II's test-retest reliability (Rosário et al., 2014). Indeed, in our MTMM analysis we found strong support for both convergent and discriminant validity in relation to time as the method. The small method effects (of multitrait monomethod correlations higher than multitrait multimethod correlations) we found were as expected, with correlations of theoretically related dimensions being high at the same measurement occurrence, particularly within some domains. We can therefore confirm Hypothesis 1b.

### Convergent and divergent validity (H2)

To test for divergent and convergent validity we correlated all COPSOQ-II scales with covariates of particular importance for school principals' occupational wellbeing, (i.e., another MTMM model), based on our theoretical review and assumed a-priori relationships. Indeed, we found all of our hypothesized convergent relationships to show the stronger correlations on average than the non-matching ones. Further, almost all of the predicted relationships reflected the highest correlation of that specific covariate with the predicted COPSOQ-II scale, thus also providing strong evidence for divergent validity.

For covariates that represented typical stress sources, one scale, however, did not show the expected relationships. Interpersonal conflicts showed its highest correlation with Role Conflicts and not as hypothesized with Trust in Management. However, Trust in Management was the second highest correlation and the difference seemed marginal.

In line with our expectations depression correlated highest with the depressive symptoms scale. Nevertheless, it showed very high correlations with various COPSOQ-II scales. The reason for this pattern might be because depression can be viewed as an outcome variable or consequence of being repeatedly exposed to stressors/demands over time, rather than being a demand itself that may or may not cause such mental health outcome (Dicke et al., 2014). Such a differentiation between stressors and strain has become standard in research on occupational wellbeing and is reflected in models such as the aforementioned JCM (Hackman and Oldham, 1976), DCM (Karasek, 1979), ERI (Siegrist, 1996), and the more recent Job-Demands Resources model (Schaufeli and Bakker, 2004; Bakker and Demerouti, 2007). Thus, a high level of depression will be related to the appearance of high levels of other strain variables (as reflected by correlations with indicators such as sleeping troubles or exhaustion which can be considered as a symptom of depression), but also with high levels of demands that caused this strain in the first place. Overall, the results reported here show that the identified principal stressors are significantly related to principals' demands and to ill-health outcomes, while autonomy and confidence are related to positive outcomes such as self-efficacy and job satisfaction. Future research should explore these sequential and causals relationships of psychosocial factors in school principals, with regard to important stress models the COPSOQ-II is based on to confirm these assumptions longitudinally. This will enable derivation of empirical and practical implications and further advance research into this red flagged occupational group.

### Individual differences due to demographic characteristics (H3)

Surprisingly our results did not reveal large differences due to demographic characteristics in the psychosocial risk factors of the school principals. In contrast to our assumptions we found inconsistent results for gender predicting ill-being as some other researchers had demonstrated as well (Friedman, 2002; Dewa et al., 2009; Darmody and Smyth, 2016). In addition, despite there only being small effects, older principals showed mostly lower levels negative risk factors and thus higher wellbeing than younger ones, while less experienced principals didn't have higher job satisfaction, but rather showed higher demands and lower wellbeing. This could indicate that individuals entering the role of school principal are more vulnerable in very early stages, but that more experience, while protective, increases skepticism about the role. This result also suggests that the increased risks to younger people in the role needs to be investigated more thoroughly as there is going to be a significant lowering of the age of principals, and previous research indicates significant increased risk for this incoming cohort (Kuper and Marmot, 2003).

In line with what we expected location and school type didn't show meaningful effects on the psychosocial risk factors in general. The strongest predictions were through working at a private rather than a public school. Indeed, working as a school principal in the private sector was predictive of many positive factors (Darmody and Smyth, 2016). This is not surprising as these school principals will have higher resources and greater autonomy. A deeper investigation of what exactly is a protective job or personal resource of these principals is important with regard to practical implications such as best practices that could be transferred to the public sector.

### School principal's risk profiles (H4)

The profiles revealed predominantly level effects with profiles from high to no risk. Here we want to discuss two risk factors that stood out, namely Job Insecurity and Family-Work Conflict.

*Job Insecurity*. Job Insecurity seems to be one of the most extreme values in all profiles and as it's direction is in line with most scales, we propose Job Insecurity to be one of the main drivers of the level of the profiles. Early on seminal work, such as Karasek et al. (1998) recognized the important role of job insecurity in the stress process. However, job insecurity has not yet been examined closely in school principals (or even teachers but see Vander Elst et al., 2014) indicating an important area for future research. It is an important factor as it is increasingly used as a new public management tool for both teachers and principals.

*Family-work conflict*. Family work-conflict (which is often confounded with work-family conflict in stress research) has been investigated with female principals (Loder, 2005), who showed that these such conflicts are an increasing problem for women administrators. Research with a more general sample of teachers can confirm that family-work and work family-conflicts predict strain and burnout (e.g., Gali Cinamon and Rich, 2010). The results presented here show an interesting pattern of two profiles were family-work conflict, but not work-family conflict, which shows a high value in the opposite direction of all other risk profiles. Put simply in Profile 3 family-work conflict shows a high-risk influence, while all other risk factors show low risk (and vice versa for Profile 5). This could indicate that for school principals in these profiles family-work conflict is involved in a trade-off. This would mean, that these principals can either have an overall low risk (high wellbeing), but this comes with the cost of a high family work conflict (Profile 3), or that they can have a good family-work balance, but that comes with the cost of an otherwise higher risk (low wellbeing). Further, the difference in the patterns for family-work and work-family conflict are another interesting area for future research, not just in the occupation of school principals.

Interestingly, Research has focused on the relationship between job insecurity and work-family conflict [which is often time confounded with family work conflict; Vander Elst et al. (2014)]. Vander Elst et al. (2014) could show a reciprocal relationship of these two risk factors for men in a sample of teachers and explain this relationship through spillover effects of job insecurity on the spouse and negative effects that impact any children. Next steps should be to investigate if the profiles are meaningful predictors of additional outcomes such as attrition or turn-over.

## Limitations and future research

As mentioned above, one limitation of the present study was that the high number of items and factors included in the COPSOQ-II lead to very complex and computationally demanding models, making standard analyses, as in our case tests for longitudinal invariance, impossible. Researchers who desire to use the instrument longitudinally should focus on a limited number of scales relevant to their research, and test invariance only for these scales. Alternatively, they can make use of the MTMM approach suggested in the present study.

Additionally, due to the nature of data collection in our study we were able to look at the correlations with our covariates only cross-sectionally. Future studies need to include longitudinal predictions and outcomes to fully explore convergent and divergent validity of the instrument. It would be of importance to continue investigating the correlations with other established questionnaires that assess work place wellbeing.

Finally, the aim of the present study was to validate the COPSOQ-II in an at risk occupational group, namely Australian school principals, to be able to provide a practical benefit for future studies considering this group. Although many studies have shown the applicability of the COPSOQ-II in other languages and cultures already, future research should test for invariance and compare the instruments properties over countries and occupations to provide evidence for the generalizability of the COPSOQ-II.

## Conclusion

The present study provides an important contribution to investigating the still understudied occupational group of school principals and validating the comprehensive COPSOQ-II instrument, considering its rapidly growing popularity with researchers and workplaces alike. The proposed factorial structure of the COPSOQ-II shows a very good fit to our large data set of school principals. In addition, the COPSOQ-II showed good support for convergent and discriminant validity over time. We found expected associations with covariates considered to be important for school principals' occupational wellbeing, which provided strong evidence for convergent and divergent validity in relation to external variables. We found overall very little effects of demographic variables in predicting the psychosocial risk factors. Working in the private sector however, seems to be beneficial for health and demands alike. School principals can be clustered in five profiles of different risk levels (from high to minimal risk). Job insecurity and family-work conflict play an important role in the formation of these profiles. Overall, the COPSOQ-II can be considered a beneficial tool for investigating psychosocial factors for school principals. Identifying these factors is of major importance, as principal's wellbeing is not only crucial in its own right, but also effects, teacher's and students' wellbeing and achievement. The present study took an important additional step in confirming a comprehensive and validated measurement instrument, thereby further closing the theory application gap and enabling the development of effective workplace interventions.

## Ethics statement

This study was carried out in accordance with the recommendations of Monash University and Australian Catholic University Human Research Ethics Committees (HREC) with written informed consent from all subjects. All subjects gave written informed consent in accordance with the HREC recommendations. The protocol was approved by the Monash University and Australian Catholic University HREC.

## Author contributions

TD was the main initiator of this research. HM supported in the application of exploratory structural equation modeling (ESEM), while PR is an expert and advised in substantive matters regarding school principals as he has been working with them for years. PP and JG both supported the lead author with the methods applied in this research. MH was involved in data preparation and substantively edited the manuscript.

### Conflict of interest statement

The authors declare that the research was conducted in the absence of any commercial or financial relationships that could be construed as a potential conflict of interest.

## Footnotes

^1^The Job insecurity scale was not collected at T2.
